# Effects of Amount and Profile of Amino Acids Supply on Lactation Performance, Mammary Gland Metabolism, and Nitrogen Efficiency in Holstein Dairy Cows

**DOI:** 10.3390/ani13111866

**Published:** 2023-06-03

**Authors:** Marina A. C. Danes, Eduardo M. Paula, Claudia Parys, Gleiciele M. Souza, João Pedro A. Rezende, Glen A. Broderick, Michel A. Wattiaux

**Affiliations:** 1 Department of Animal Science, University of Lavras, Lavras 37200-900, MG, Brazil; gleiciele.souza3@estudante.ufla.br (G.M.S.); joao.rezende5@estudante.ufla.br (J.P.A.R.); 2 Institute of Animal Science, Beef Cattle Research Center, Sertãozinho 14160-970, SP, Brazil; emarostegandepaula@gmail.com; 3 Evonik Operations GmbH, 63457 Hanau-Wolfgang, Germany; claudia.parys@evonik.com; 4 Broderick Nutrition and Research LLC, Madison, WI 53705, USA; gbroderi@wisc.edu; 5 Department of Animal and Dairy Sciences, University of Wisconsin, Madison, WI 53706, USA; wattiaux@wisc.edu

**Keywords:** dairy cows, protein, amino acids, nitrogen efficiency, mammary gland metabolism

## Abstract

**Simple Summary:**

Efficiency of nitrogen utilization (ENU) for productive purposes is low (20–30%), partially due to post-absorptive amino acid (AA) catabolism. Catabolism can be reduced by increasing mammary AA removal from the blood, allowing less AA to reach non-mammary tissues. Reducing the AA supply did not decrease milk protein yield and increased ENU, while altering AA profile did not affect ENU. Infusing AA into a low protein diet increased energy corrected milk due to changes in mammary metabolism. Such response seemed to be not only due to substrate effect but also to the regulatory role of the infused AA.

**Abstract:**

To evaluate the effects of amount and profile of amino acid (AA) on milk protein yield (MPY), mammary metabolism, and efficiency of nitrogen use (ENU), ten cows were used in 5 × 5 replicated Latin squares and fed a positive control (16.1% crude protein-CP) or two lower CP diets (14.6 and 13.2%) with or without essential AA (EAA) infusion. The EAA solutions provided predicted limiting EAA in each treatment and were continuously infused into the abomasum of the cows. Milk production and MPY were not affected by treatment (mean 35.4 kg/d and 1.03 kg/d, respectively). Efficiency of nitrogen utilization was increased as dietary CP decreased but was not affected by EAA infusion (*p* < 0.01). Energy-corrected milk production was increased by EAA infusion into 13.2% CP, but not into 14.6% CP diet (*p* = 0.09), reaching the positive control value. Infusions increased mammary affinity for non-infused EAA (Ile, Phe, Thr, and Trp), allowing the same MPY despite lower arterial concentrations of these AA. Higher arterial concentrations of infused EAA did not increase their mammary uptake and MPY (*p* = 0.40; *p* = 0.85). Mammary metabolism did not fully explain changes in N efficiency, suggesting that it might be driven by less extramammary catabolism as AA supply was reduced.

## 1. Introduction

Ruminants are intrinsically less efficient than non-ruminants in using dietary nitrogen (N) to productive purposes due to inevitable N losses throughout the metabolism of dairy cows [1]. A classic review of the literature reported that the efficiency of N use (ENU) in dairy cows was only 24.7% in North American and 27.7% in North European herds [2]. An important portion of the inefficiency comes from post-absorptive metabolism, specifically amino acid (AA) catabolism by splanchnic and peripheral tissues [3]. Since most of nutrient supply to any tissue comes from arterial blood, the amount of AA that reaches the non-mammary tissues and are, therefore, available to catabolism, depends on how much is released by the mammary gland [4,5]. Therefore, nutritional strategies that stimulate mammary gland removal of AA from arterial supply have the potential to increase ENU.

Until recently, the efficiency with which mammary gland uses AA to synthesize milk protein was considered fixed and equal for all the AA by the main nutritional models used to formulate dairy diets [6]. However, substantial scientific evidence was available in the literature demonstrating that this was not the case, and that the efficiency varies with the supply of AA [7], supply of energy [8], and hormone levels [9]. In fact, reducing dietary crude protein (CP) content is an effective strategy to increase ENU, but it can impair milk protein yield [10] due to reduced feed intake or deficiency of specific AA.

Additionally, the profile of AA supplied to the mammary gland may also affect the efficiency of their use for protein synthesis [11]. In theory, the closer the supplied AA profile is to the profile of the protein synthesized in the target tissue, the higher the efficiency of AA utilization should be. Swine and poultry nutritionists have adopted this approach in their ration formulation, feeding low CP diets balanced with specific EAA to better match the animal requirements, resulting in gross N efficiencies of 40% or greater [12]. In lactating dairy cows, Haque et al. [11,13] adjusted the AA profile of low crude protein diets to match an ideal profile and observed increases in ENU.

The variable efficiency of AA used to synthesize milk protein is a consequence of a highly flexible mammary metabolism. The mammary gland can adjust its AA uptake, as well as its local blood flow, to match the demand for specific precursors [14]. However, the responses to varying dietary AA supplies are not consistent. For instance, the mammary plasma flow of dairy cows was increased by 32% when the CP content of a diet was reduced from 16.5 to 12% [15], while it was not altered when either a 15 or 12% CP was fed [16]. Likewise, the mammary plasma flow of dairy cows was not altered by abomasal casein infusion [8], but it was numerically decreased by 31% when a complete EAA mixture was abomasally infused on top of a 14% CP diet [17]. A better understanding of how dietary manipulations related to AA supply affect mammary metabolism might provide insights to formulating diets that allow for greater ENU.

Therefore, the objectives of this study were to expose the mammary gland to different amounts and profiles of metabolizable AA and evaluate: (1) the response of milk protein yield and mammary metabolism in terms of mammary plasma flow, AA clearance rate, and the ratio of mammary AA uptake to milk AA output; (2) how the mammary gland responses affect the efficiency of metabolizable AA utilization for milk protein yield and overall dietary N efficiency. The hypothesis of this study was that the efficiency with which the mammary gland uses plasma AA to synthesize milk protein is variable and depends on the amount and profile of AA reaching the mammary gland.

## 2. Materials and Methods

### 2.1. Experimental Procedure

Ten multiparous Holstein cows, fitted with permanent 10 cm rumen cannulas (Bar Diamond Inc., Parma, ID, USA), averaging (mean ± SD) 141 ± 44 DIM, 44.5 ± 8.8 kg of milk/d, and 682 ± 61 kg of BW at the start of the experiment were used. Cows were grouped by DIM into two 5 × 5 Latin squares and, within square, were randomly assigned to one of five treatment sequences, balanced for residual effects. Each 14-d experimental period included 10 days for adaptation to the treatments and 4 days for data collection. Cows were housed in tie stalls with free access to fresh water. All cows were injected with bST (500 mg of Posilac; Elanco Animal Health, Greenfield, IN, USA) every other week on the sixth day of each experimental period. Animal care and experimental procedures were approved by the Animal Care and Use Committee of the College of Agriculture and Life Science of UW-Madison (Research Animal Resources Center protocol # A-07-3400-A00286).

The experimental diets were fed as total mixed ration (TMR) and composed of alfalfa silage, corn silage, high-moisture shelled corn (HMSC), solvent-extracted soybean meal (SBM), solvent-extracted canola meal (CM), soybean hulls, and a mineral/vitamin premix. The nutritional composition of the principal feed ingredients is presented in Table 1.

The positive control treatment was formulated to meet the protein requirements [6] of the average cow at the beginning of the trial (25.8 kg DMI, 45 kg milk/d, 3.6% fat, 2.8% true protein), with 16.5% CP (dry matter-DM-basis). Two other diets with lower CP levels were formulated with the objective of reducing AA supply to the animal. Reduction of diet CP to 15.0% and 13.5% of DM was achieved by replacing SBM and CM with soybean hulls and HMSC in a proportion of approximately 1:4 (HMSC: soybean hulls, DM basis; Table 2). In addition, in order to manipulate the profile of AA supply, the two lowest CP diets were fed with or without a continuous abomasal infusion of a mix of all limiting essential AA (EAA). Amounts of AA (g/d) infused into the abomasum of cows for each treatment were 7 and 16 for L-His, 6 and 28 for L-Leu, 15 and 38 for L-Lys, 8 and 13 for DL-Met, 0 and 5 for L-Val, and 36 and 99 for the sum of all AA for diets formulated for 15.0 and 13.5% CP, respectively. These amounts were computed for each diet according to the AA balance (i.e., the AA with negative balances) at the beginning of the trial using AminoCow Dairy Ration Evaluator, version 3.5.2 (Evonik Operations GmbH, Hanau-Wolfgang, Germany).

Therefore, five treatments were constructed: (1) 16.5% CP diet—positive control; (2) 15.0% CP diet with EAA infusion; (3) 15.0% CP diet without EAA infusion; (4) 13.5% CP diet with EAA infusion; and (5) 13.5% CP diet without EAA infusion. The infusion solutions were prepared every other day by dissolving the appropriate amount of each AA (provided by Evonik Operations GmbH, Hanau-Wolfgang, Germany) in warm tap water. The AA composition of the two solutions (one for each diet CP level) was determined at the beginning of the experiment and maintained constant throughout the trial. Solutions were continuously infused with a peristaltic pump into the abomasum through a tube connected at one end to the rumen cannula and at the other end to a flexible plastic flange that anchored the infusion line in the abomasum [18]. The tube was passed through the reticulo-omasal and omasum-abomasal orifices using the polyvinyl chloride pipe insertion tool described by Gressley et al. [18]. Solution bottles were weighed every day to measure the exact amounts of EAA infused.

During adaptation (d 1 to d 10), cows were fed once daily at about 1000 h. During the last 4 days of each period, cows were fed four times a day, at 04:00, 10:00, 16:00, and 22:00 h, to minimize post-prandial variation of blood metabolite concentrations. Feed offered was adjusted daily to yield 5 to 10% orts. Weekly composites of the ingredients, TMR and orts were obtained from daily sub-samples. Dry matter content was determined in weekly composites of alfalfa silage, corn silage, HMSC, TMR, and orts by drying at 60 °C for 48 h. The DM contents of the feed ingredients were used to adjust as-fed composition of TMR every week over the trial. Dry matter intake was computed based on the difference between feed offered and feed refused and the 60 °C DM determinations of weekly composites of TMR and orts. Dried (60 °C) samples of the two silages, HMSC, and undried samples of SBM, CM, and soybean hulls from week 2 of each period (5 samples of each ingredient over the trial) were ground to pass a 1 mm screen (Wiley mill, Arthur H. Thomas, Philadelphia, PA, USA). Ground samples were analyzed for DM at 105 °C [19] (method 967.03), total N (Leco 2000 N Analyzer; Leco Instruments, Inc., St. Joseph, MI, USA), ash and OM [20], sequentially for neutral detergent fiber (NDF), acid detergent fiber (ADF), and acid detergent insoluble nitrogen (ADIN) [21] using heat-stable amylase and sodium sulfite [22] and for neutral detergent insoluble nitrogen (NDIN) without the α-amylase and sodium sulfite during extraction [23]. Dietary ingredients were also analyzed for AA, including performic acid oxidation of Met and Cys, conducted by Evonik Operations GmbH [24,25].

Cows were milked twice daily at 05:00 and 17:00 h and milk weights were recorded at each milking. Milk samples were collected from both a.m. and p.m. milkings during the last 4 days of each experimental period, preserved with 2-bromo-2-nitropropane-1,3-diol, and analyzed for fat, true protein, lactose, and milk urea nitrogen (MUN) content by mid-infrared spectroscopy (AgSource Laboratory, Verona, WI, USA) using a Foss FT6000 (Foss North America Inc., Eden Prairie, MN, USA; [19] (method no. 972.16). Concentrations and yields of fat, true protein, lactose, and MUN concentration, were computed as weighted means based on a.m. and p.m. milk yields on each test day. Yields of energy-corrected milk (ECM) were computed by the energy output in milk (NEL, Mcal/d = milk yield, kg/d × ((0.0929 × percent fat) + (0.0563 × percent true protein) + (0.0395 × percent lactose)); [26] and the assumed energy content of 4% fat corrected milk of 0.749 Mcal of NEL/kg [27].

Spot urine and fecal samples were also collected on day 13 of each period. Urine was collected at 01:00, 07:00, 13:00, and 19:00 h, and feces were collected at 01:00 and 13:00 h. Urine samples (15 mL) were acidified with 60 mL of H_2_SO_4_ solution (0.072 N) and analyzed for total N by elemental analysis (Leco FP-2000 N Analyzer) for urea using an automated colorimetric assay [28] adapted to flow injection (Lachat Quick-Chem 8000 FIA; Zellweger Analytical, Milwaukee, WI, USA), and for creatinine (Lachat QuikChem 27-227-00-1-A). Daily urine volume was estimated using creatinine as a marker, assuming a creatinine excretion rate of 29 mg/kg of BW [29]. Urinary allantoin [30] and uric acid (kit no. 1830, Thermo DMA, Waltham, MA, USA) concentrations were also determined in urine using assays adapted to a 96-well plate reader. Daily excretions of urea N, total N, allantoin, and uric acid were computed from daily urine output and mean urinary concentrations in each period. Microbial protein yield was estimated from the daily excretion of purine derivatives (allantoin + uric acid), according to Chen and Gomes [31], except that absorbed purine was computed using a regression equation [32]. Fecal samples were dried at 60 °C for 72 h and ground through a 1 mm screen (Wiley mill). Equal amounts of DM from a.m. and p.m. samples from each cow in each period were composited and analyzed for DM (105 °C), ash, OM, NDF, ADF, and N as described earlier for feeds. Daily fecal output and total tract apparent digestibility of nutrients was estimated using the indigestible ADF content (ADF remaining after 12-d in situ incubations) in feces and TMR as an internal marker.

Blood samples were taken on the last day of each period into heparinized test tubes, simultaneously from a coccygeal vessel and the subcutaneous abdominal vein, approximately at 06:00 (right after a.m. milking), 09:30, 13:00, and 16:30 h (right before p.m. milking). The composition of coccygeal blood was assumed to be equivalent to that of arterial blood [33]. Samples were centrifuged (1500× *g*, 4 °C, 15 min), and the resulting plasma was analyzed for urea-N by incubation with urease followed by phenol-hypochlorite colorimetric assay [34] and for free AA. Amino acid analysis was performed by isotope dilution [35] using a gas chromatograph coupled with a mass-spectrometer (GC-MS; single quadrupole GCMS-QP2010, Shimadzu, Tokyo, Japan). Infusion solutions collected during week 2 of each period were also analyzed for AA in the same manner as plasma samples.

Mammary metabolism was evaluated based on responses to treatments in mammary plasma flow, mammary uptake/release (net flux) of individual AA, clearance rate, and the ratio of mammary AA uptake to milk AA output. Plasma flow across the mammary gland was estimated according to the Fick principle using Phe + Tyr as internal markers, assuming a 3.5% contribution from blood-borne proteins [36]: mammary plasma flow (L/h) = (milk secretion of Phe+Tyr (umol/h) × 0.965)/(arterial-venous concentration difference of Phe+Tyr (μmol/L)) [37], based on milk protein yield of the milking immediately following the blood sampling. Net fluxes of AA across the mammary gland (mammary uptake/output) were calculated for each cow within each period as the product of mammary plasma flow and average plasma arterial-venous AA concentration difference. The clearance rate represents the ability of the mammary gland to clear AA from the plasma per unit of time [38]. The clearance rate was computed for each AA with the model proposed by Hanigan et al. [38]: clearance rate (L/h) = [(AA arterial-venous difference) × mammary plasma flow]/AA venous concentration. Milk AA output used to compute the ratio of mammary uptake to milk output was calculated using milk protein yield measured on the milking immediately following the blood sampling, with a 3.5% correction for blood-borne proteins [36], and AA composition of milk protein as reported in Lapierre et al. [37].

Observed animal inputs (DMI, milk yield and composition, DIM, and BW), analyzed the nutrient composition of feed ingredients, and amounts of each EAA infused (based on measured weights of infused solutions and analyzed AA composition) were used to estimate [39], for each cow/period, the dietary CP, rumen-degraded protein (RDP) and rumen-undegraded protein (RUP) contents, MP supply and balance (g/d), NEL supply and balance (Mcal/d), digestible EAA flows (g/d), individual AA efficiencies, and milk protein yield.

### 2.2. Statistical Analysis

Dry matter intake, milk and components yield, and milk composition were averaged over the last 4 days of each period. Plasma concentrations of AA were averaged over the four sampling times on d 14 of each period, except for Lys, His, Asn, and Trp, for which only one composite sample was analyzed, and the result was used directly in the data set. Urine variables (total N, urea N, purine derivatives) were averaged over the four sampling times on d 13 of each period. Because fecal samples were composited prior to analysis, variables originating from their analyses had only one observation per cow/period; NASEM [39] estimates also had only one data point per cow/period.

Data were analyzed using the Mixed procedure of SAS (SAS Institute Inc., Cary, NC, USA; 2013) for a replicated 5 × 5 Latin square. Cow × period was the experimental unit. One cow that started the trial injured her neck in a parlor gate and was replaced with a spare cow from period 3 on; therefore, her data from periods 1 and 2 were missing. Furthermore, due to a problem with infusion lines and illness, data from 3 cows during period 5 were discarded. Over the trial, there were 10 observations for treatment 1, 9 observations for treatments 3, 4, and 5, and 8 observations for treatment 2.

The model for all variables except rumen traits included the fixed effects of square, period, and treatment and the random effect of cow within square. Data were considered outliers and excluded from the data set when a Student’s residual higher than 3 or lower than −3 was detected. Significance was declared at *p* ≤ 0.10. When the main effect of treatment was significant, LSD was used to separate means at *p* ≤ 0.05. Least squares means with the highest SEM are reported for all data.

NASEM [39] predictions of individual milk protein yield were compared with observed yield with the REG procedure of SAS, and the model accuracy was assessed using the coefficient of determination (R^2^) and the root mean square error (RMSE) of a linear regression between actual and predicted milk protein yield.

## 3. Results

### 3.1. Diets and Infusion Solutions

Diet ingredients and nutrient compositions are presented in Table 2. As dietary CP level was decreased, NDF and starch were increased. Diet CP levels were lower than formulated due to differences in the nutrient composition of feed ingredients. However, the target interval of 1.5 percentage units in diet CP was maintained.

Table 3 presents the NASEM [39] estimates of protein and energy balances, as well as the duodenal supply of AA, based on measured and analyzed inputs for each individual experimental unit (cow × period). Energy balance did not differ (*p* = 0.89) among treatments, and it was positive for all of them. Dietary CP levels affected (*p* < 0.01) RDP contents, reaching levels below 10% DM in the lower CP diets. The AA infusions increased diet RUP levels (*p* < 0.01) relative to the non-infused treatments but not as much as to the positive control. Metabolizable protein supply was reduced only by the lowest CP diet without infusion (*p* < 0.01) compared to the positive control. According to NASEM [39], all treatments were MP deficient, ranging from 2.4% deficient in the 16.1% CP diet to 16.2% deficient in the non-infused 13.2% CP diet.

The estimated duodenal flow of EAA, in g/d, was affected by treatment (Table 3, *p* < 0.01). Overall, among the non-infused treatments, estimated AA flows were decreased by 13.2% CP relative to the positive control. Additionally, the duodenal supply of only some of the infused AA were increased (relative to the non-infused diet) due to numeric differences in DMI. His and Met were increased in both treatments, while Leu and Val were not altered in either. The lys flow was only increased in the 13.2% CP diet. As expected, the efficiency use of all the AA were also affected by treatment (Table 3, *p* < 0.01). Overall, the greater the supply of an AA, the lower its efficiency. In agreement with the negative MP balances for all treatments, most of the efficiencies were higher than the target efficiency recommended by NASEM [39].

### 3.2. Animal Performance

Dry matter intake, milk yield and composition, and plasma urea N (PUN) are presented in Table 4. The average milk yield across all treatments throughout the trial was approximately 10 kg/d less than that for which the diets were formulated. This was at least partly a consequence of the strong heat stress experienced by the cows during the study. In fact, in an experiment that reported mean THI values of 71 and 87 for the control and heat-stressed group, respectively, heat stress was associated with lower rumen pH and changes in rumen microbiota and metabolism, with more lactate and less acetate-producing species in the population, which probably negatively affected milk production [40]. Furthermore, heat stress in our experiment probably reduced DMI, which may have reduced microbial protein synthesis and impacted AA available for the cow. However, this effect was probably the same for all treatments and did not affect the comparison among them. Milk yield and DMI were unaffected by the treatments (*p* > 0.10). The numerical differences in DMI between the infused and non-infused treatments within dietary CP levels explain part of the differences in EAA supply described in Table 3. There was no effect of treatment (*p* > 0.10) on milk protein and lactose content or yields. However, the highest milk fat yield (kg/d) and concentration were observed for the 13.2% CP diet with EAA infusion. Fat yield increased (*p* < 0.05) relative to the non-infused 13.2% CP diet, the infused 14.6% CP diet, and even to the positive control, and fat concentration increased (*p* < 0.05) relative to the two highest CP diets. The yield of ECM was affected by treatment (*p* = 0.09), with a decrease for the 13.2% CP diet without infusion and for the 14.6% CP diet with infusion, relative to the other three treatments (*p* < 0.05). Milk urea nitrogen responded as expected and decreased (*p* < 0.05) with the reduction in dietary CP. Infusion of AA reduced MUN on the 14.6% CP diet but not for the 13.2% CP diet. Similarly, PUN was decreased (*p* < 0.05) by the reduction in diet CP but was not affected (*p* > 0.05) by AA infusion.

Nutrient digestibility and intake of digestible nutrients are presented in Table 5. Dry matter digestibility was affected by treatment (*p* < 0.10), and it was greater in the two 13.2% CP diets than in the 16.1% CP diet (*p* < 0.05). However, digestible DMI was not affected by treatment (*p* > 0.10). Greater DM digestibility for the 13.2% CP diets was likely brought about by the higher inclusion of soybean hulls in these diets, which is suggested by the 7 percentage-units greater NDF digestibility for the lowest CP diet than the positive control (*p* < 0.05). As a result, intake of digestible NDF was increased (*p* < 0.05) as dietary CP decreased but was not affected by AA infusion, which was expected since the infusion was post-ruminal. There was no effect of treatment (*p* > 0.10) on organic matter (OM) or CP digestibility and on OM intake. As expected, digestible CP intake was affected by the treatment, but it was only a significant difference between 13.2% CP diets and 16.1% CP diets (*p* < 0.05). Microbial protein yield was estimated from daily excretion of urinary purine derivatives (Table 5) and was unaffected by treatment (*p* > 0.10).

Nitrogen balance is presented in Table 6. Nitrogen intake differed among the dietary CP levels (*p* < 0.05), as expected. The EAA infusion added, on average, 6.3 g/d of N for the 14.6% CP treatment and 16.3 g/d of N for the 13.2% CP treatment. The remaining numerical differences in N intake between the two treatments within the CP level are due to the non-significant differences in DMI since diet composition was the same. Treatments did not affect milk N (milk true protein/6.38) secretion (*p* > 0.10), which, coupled with the reduction in N intake, caused the efficiency of N use (ENU, milk N/N intake) to increase as dietary CP decreased (*p* < 0.05). Within the dietary CP level, however, AA infusion did not change ENU (*p* > 0.05). The main route of excess N excretion is urine, specifically in the form of urea N. Accordingly, decreasing dietary CP reduced (*p* < 0.05) total N concentration in the urine and daily urinary N excretion. Urinary urea N as a proportion of total urinary N decreased (*p* < 0.05) from 94.1% in the 16.1% CP diet to an average of 81.8% in the 14.6% CP diets and 72.4% in the 13.2% CP diets. Once again, AA infusions did not affect any of the urinary N variables (*p* > 0.05). Fecal N concentration and excretion were not affected by treatment (*p* > 0.10), and excretion averaged 33.5% (SD 1.4) of N intake. The sum of the proportions of N intake secreted in the milk and excreted in feces and urine should be close to 100% since N retention in lactating mature dairy cows is expected to be minimal. However, unaccounted N (100 − (milk N/NI) − (urine N/NI) − (fecal N/NI)) varied from 14.6 to 20.5% are greater values than expected. Even though there was no treatment effect for unaccounted N (*p* > 0.10), there was a numerical decrease as the diet CP decreased and a numerical increase with the AA infusion when compared to within diet CP level.

### 3.3. Mammary Metabolism

Plasma arterial concentration of total AA, accounted on an N basis (TAA-N), was not affected by the treatments (*p* > 0.10, Table 7) and averaged 2400 μmol of N/L of plasma (SD 78). The concentration of NEAA, on an N basis (NEAA-N), was also not affected by treatments (*p* > 0.10) and averaged 1388 μmol of N/L of plasma (SD 61). However, decreasing diet CP from 16.1 to 13.1% without EAA infusion decreased (*p* < 0.05) arterial N concentration from EAA (EAA-N). Infusion of EAA to the 13.2% CP diet increased (*p* < 0.05) EAA-N to the same level as the positive control and the infused 14.6% CP treatment and higher than the 14.6% CP diet without infusion (*p* < 0.05). Within the EAA, the arterial concentration of N from EAA from group 1 (Phe+Tyr, His, Met, Thr, and Trp) was increased by EAA infusion in the 13.2% CP diet relative to the non-infused treatment (*p* < 0.05), while diet CP levels did not affect group 1 AA-N concentration (*p* > 0.10). Group 2 AA-N (N from Ile, Leu, Lys, and Val) concentration was decreased for the infused 14.6% CP diet and the non-infused 13.2% CP diet relative to the positive control (*p* < 0.05), even though Lys and Leu were supplied in the infusate supplementing the 14.6% CP diet. In fact, the EAA infusion did not affect group 2 AA-N in any CP level relative to their iso-CP non-infused treatment (*p* > 0.10).

The increased EAA-N concentration in the infused 13.2% CP treatment was brought about by increases in Lys, Met, and His concentration relative to the non-infused 13.2% CP diet (*p* < 0.05). While Lys was increased to the same level as the positive control, His and Met concentrations were higher for the infused 13.2% than for the 16.1% CP. For the 14.6% CP diet, infusion increased Met to levels higher than the three non-infused treatments but lower than the infused 13.2% CP diet (*p* < 0.05), while His was increased to the positive control level (*p* < 0.05). Lysine concentration was not affected by CP level (*p* > 0.05) or EAA infusion to the 14.6% CP diet (*p* > 0.05), but it was increased by the infusion in the 13.2% CP level (*p* < 0.05). Leucine was not affected by treatment (*p* > 0.10) even though it was infused in both dietary CP levels. Valine and Ile were decreased in the 13.2% CP diets and in the infused 14.6% CP diet, relative to the positive control (*p* < 0.05). Interestingly, the EAA that were not infused in any treatment (Phe, Thr, and Trp) were numerically decreased (*p* > 0.10) in the infused treatments. Among the NEAA, Ser and Tyr concentrations were decreased (*p* < 0.05) by the infusion. Tyrosine was not affected by CP levels (*p* > 0.05), but Ser concentration was higher in the non-infused 13.2% CP diet than in the non-infused 14.6% CP diet (*p* < 0.05). Similarly, Gly concentration was the highest in the non-infused 13.2% CP treatment (*p* < 0.05). The infusion to the 13.2% CP diet increased (*p* < 0.05) Cys concentration relative to the non-infused 13.2 and 14.6% diets, likely due to the greater supply of Met in this treatment.

Mammary plasma flow was not affected (*p* > 0.10, Table 8) by treatments and averaged 832 L/h (SD 22). With no effects on AVD or mammary plasma flow, mammary gland net flux also was not altered by the treatments (*p* > 0.10, Table 8) for all AA but Gln. Mammary Gln uptake was increased in the infused 13.2% CP diet compared to the positive control (*p* < 0.05), while the other three treatments were intermediate. Mammary Gly flux was not different from zero (*p* > 0.05) in any treatment.

The mammary clearance rate was affected (*p* < 0.10) by treatments for His, Met, Phe, Ser, Thr, and Tyr in different manners (Table 9). The non-infused 13.2% CP diet had the highest clearance rate for His, and it was different (*p* < 0.05) from the infused 14.6% CP diet, which had the lowest His clearance rate. The clearance rate for Met was decreased in the two treatments with EAA infusion relative to the other three (*p* < 0.05). Phenylalanine and Ser clearance rates were increased for the infused 13.2% CP diet compared to the three non-infused treatments (*p* < 0.05). The non-infused 13.2% CP diet had a Thr clearance rate lower than all the other treatments (*p* < 0.05), while the infused 13.2% CP diet had a Tyr clearance rate greater than the other treatments (*p* < 0.05). The clearance rate for total AA-N, EAA-N, NEAA-N, group 1 AA-N, and group 2 AA-N were not affected by the treatments (*p* > 0.10).

The ratio between mammary AA uptake and milk AA output was affected by treatment only for Met (*p* = 0.07) and Ser (*p* = 0.03; Table 10). For Met, the ratio was decreased in the two non-infused diets relative to the positive control (*p* < 0.05). For Ser, the ratio was greater in the infused 13.2% CP treatment than in the non-infused 14.6% and non-infused 13.2% CP diets (*p* < 0.05). Besides the treatment comparison, each individual ratio was also compared to unity to allow speculation about the intra-mammary metabolism of each AA. Ratios greater than one indicate that the AA was used for other functions besides milk protein synthesis (i.e., synthesis of NEAA, energy production). On the other hand, ratios smaller than one indicate that the part of the AA amount secreted in milk was synthesized in the mammary gland, in addition to what was taken up by the mammary gland from the circulation. Ratios equal to one indicate no intra-mammary AA metabolism. For all treatments, mammary AA uptake to milk AA output ratio was not different from one (*p* > 0.10) for Glu, Thr, and Trp, was greater than one (*p* < 0.05) for Ala, Ile, Leu, and Phe, and was smaller than one (*p* < 0.05) for Asn, Asp, Pro, Ser, and Tyr. For Lys and Val, the ratios were greater (*p* < 0.10) in all treatments but the infused 14.6% CP diet. Methionine and His ratios were greater than one (*p* < 0.05) for the positive control. Cysteine ratio was greater than one (*p* < 0.05) for the non-infused 14.6% CP diet, while Gln ratios were smaller than one (*p* < 0.05) for the positive control and the two non-infused diets and not different from one (*p* > 0.10) in the two infused treatments.

NASEM [39] predictions for milk protein yield were evaluated against the observed production, resulting in an equation (predicted vs. observed) with R^2^ of 0.71 and a RMSE of 117.8 g/d. The same exercise was performed with NRC [6] and the resulting equation had an R^2^ of 0.59 and an RMSE of 138.8 g/d. On average, cows produced 1015.8 ± 216.2 g/d of milk protein. NASEM [39] predicted that cows would produce 1047.7 ± 141.5 g/d, while NRC [6] predicted that cows would produce 873.3 ± 158.3 g/d.

## 4. Discussion

### 4.1. Animal Performance

To test our hypothesis, we designed a diet with sufficient amounts of MP as a positive control, two diets with increasing levels of MP deficiency as negative controls, and two treatments that abomasal-infused back to the negative controls the limiting AA, therefore correcting the AA profile of the reduced MP. According to NASEM [39] estimates with actual inputs from the trial, cows were marginally MP deficient on the positive control (2.4% of requirements, −51 g/d), and the reduction in dietary CP exacerbated the deficiency to 7.4% (−154 g/d) and 16.2% (−337 g/d) of requirement. Surprisingly, milk and milk protein yield were unaltered by the treatments.

The negative effects of low CP diets on milk yield are usually associated with decreases in DMI [11,41,42]. In fact, this was a concern in the lowest CP diets since their RDP contents were below 10%, the minimum recommended by NASEM [39] to avoid impairment of microbial yield, fiber degradation, and DMI. Reductions in NDF digestibility have been observed in diets with negative RDP balance with [41] or without [43] decreasing DMI. Lee et al. [41], in a 10-week continuous trial, fed a 15% deficient MP diet (MP balance −317 g/d, [6]) and, in contrast to our results, observed a decrease in milk and milk protein yield. However, they also observed a decrease in DMI, which was likely the cause of their results. On the other hand, Lee et al. [44] did not observe any effect of a 10% MP deficient diet (−256 g/d, [6]) on DMI, nutrient digestibility, milk, and milk protein yield, in Latin square experiment with 21-d periods. The duration of the deficiency might play a role in DMI response; thus, results from short-term studies should be interpreted with caution.

In the present study, most of the protein supplements were replaced by highly fermentable NDF from soybean hulls, which likely resulted in the increase in NDF digestibility observed in the 13.2% CP diets. Moreover, cows were fed four times/day during the sampling period, which provided smaller amounts of feed every 6 h and might have slowed down the passage rate and increased digestibility. Additionally, N recycling from the blood to the rumen is known to increase as N intake is reduced [45], suggesting that a deficiency of RDP could have been partly corrected by N recycling.

Besides the effect on DMI, reducing dietary CP levels might decrease milk protein yield due to a smaller MP supply to the mammary gland. Negative effects on milk protein yield of cows with production levels similar to the present study were observed when dietary CP levels were reduced from 16.9 to 15.0% [46], despite similar DMI. On the other hand, Olmos Colmenero and Broderick [10] did not observe a significant decrease in milk protein yield when dietary CP was reduced from 16.5 to 15.0 and 13.5%, levels similar to those fed in the present study. However, these trials did not report MP balance, making it more difficult to interpret and compare results.

Although not statistically significant, we observed that the 13.2% CP treatment with EAA infusion produced 84 g/d more protein than the non-infused 13.2% CP diet, which represented 59.6% recovery of the extra MP supply this treatment received (99 g from the infusate and 57 g from a numeric increase in DMI). Danes et al. [8] observed recoveries ranging from 13.5 to 47% when casein was infused alone or with an energy source. This very high marginal response confirms the deficiencies of such AA in the diet and suggests the potential for AA balancing as a strategy for ration formulation.

On the other hand, the strategy was not successful for the 14.6% CP diet, and the milk protein yield was 13 g/d smaller for the infused treatment (not significant). However, the MP supply in the infused 14.6% CP treatment was the same as for the non-infused diet, despite the infusion of 36 g/d of EAA, due to a numeric decrease in DMI. Still, even with the same MP supply, the infused treatment had a better AA profile, with greater contents of Met, Lys, and His and an adequate Lys:Met ratio [6] than the non-infused diet.

The lack of response to a better AA profile in the 14.6% CP diet suggests that the positive effect of AA infusion in the 13.2% CP treatment was not due to the supply of an adequate substrate. Amino acids can also function as regulators of metabolic pathways, such as protein synthesis. Essential AA have been shown to stimulate mTORC1 phosphorylation in bovine mammary tissue slices [47], and these changes were correlated with changes in rates of casein synthesis in mammary tissue slices [48]. Methionine and Leu have known stimulatory roles on mTORC1 pathway [9,49,50] and both were infused in much greater amounts into the 13.2% CP diet than into the 14.6% CP diet. Additionally, the 13.2% CP treatment provided more energy than the 14.6% CP diet, due to greater concentration of carbohydrates and numeric greater DMI. Energy supply, especially from glucogenic sources, can also stimulate milk protein synthesis [8] and make the synthetic machinery more responsive to other stimuli. Pszczolkowski et al. [9] recently demonstrated that insulin is required for EAA to stimulate mTORC1 activity in MAC-T cells. Even though insulin was not measured in our study, greater starch supply in the 13.2% CP diet should have increased insulin and may have made the mammary gland more responsive to the AA infused.

The milk protein prediction equation of NASEM [39], in fact, includes digestive energy intake, as well as supply of five individual EAA (His, Met, Leu, Ile, and Lys), recognizing both substrate and regulatory effects. Such an approach is a much better representation of mammary gland biology than previously assumed in NRC [6], in which milk protein yield was an exclusive function of MP supply. This was confirmed by our comparison between the two models [6,39]. The NRC [6] approach could never predict an increase in milk protein yield due to energy supply or specific AA that increases MP amounts minimally.

An unexpected response to the infusion on the 13.2% CP diet was the significant increase in milk fat yield. Cows fed the 13.2% CP with EAA infusion treatment secreted on average 154 g/d more fat than the non-infused 13.2% CP diet and the positive control. The increase over the positive control could have been attributed to the higher NDF digestibility in the 13.2% CP diets. However, the same NDF digestibility was observed for both 13.2% CP treatments, and the non-infused one did not change milk fat yield relative to the positive control, suggesting that the EAA infusion to this diet had some additional effect on milk fat secretion. Part of the effect was likely a consequence of the numerical increase of 0.8 kg/d DMI for the infused treatment. However, post-ruminal metabolism of AA and fatty acids share a number of pathways that could also be responsible for the response in fat yield with EAA infusion.

Excess glucogenic AA (Met, His, and Val) could potentially have spared glucose and increased its availability to the mammary gland [51]. Glucose is used to synthesize lactose but also provides reducing equivalents necessary for fatty acid de novo synthesis in the mammary gland through the pentose phosphate cycle. Additionally, lactose yield was also the highest in the infused 13.2% CP treatment. Moreover, individual AA can directly affect fat synthesis. The most studied AA is Met, which through the one carbon (C) cycle, transfers its methyl group to phosphatidylcholine [52]. Phosphatidylcholine is part of very low-density lipoproteins that transport long-chain fatty acids from the liver to the peripheral tissues in ruminants, including the mammary gland. The increase in milk fat yield with Met supplementation has been observed in other studies [53,54]. Another direct link occurs with the ketogenic AA that can be incorporated directly into acetyl CoA. The two exclusively ketogenic AA Leu and Lys were both supplied in the infused 13.2% CP treatment, and Lys plasma concentration was the highest for this treatment. A third connection between AA and fatty acid metabolism at the cell signaling level has been reported. Li et al. [55] studied the effects of different EAA profiles on lipogenic gene network expression in bovine mammary cells in vitro and observed a potentially important role of EAA ratios in the coordination of milk fat synthesis via the transcription factors PPARG and SREBF1. Besides milk fat yield, the regulatory effect of AA help explains the numeric increases in protein and lactose yields, which resulted in greater ECM for the infused 13.2% CP diet.

Because dietary CP was decreased and milk protein yield remained unchanged, the efficiency of utilization of dietary N to synthesize milk protein N was increased as dietary CP decreased. The reduction in dietary CP is the most effective way to increase ENU and reduce N excretion to the environment [2,56]. The better utilization of N as dietary CP decreased was also reflected in MUN and PUN values, which were reduced on the lowest CP diet.

The concept of an EAA profile ideal for milk protein synthesis has been proposed in the literature using different methodologies, including dose–response experiments and meta-analysis [6,7,57]. To test that, two trials supplied an ideal EAA profile (according to Doepel et al. [7] and Rulquin et al. [57]) through diet and abomasal infusion of AA at two dietary MP levels [11,13]. In both trials, regardless of the dietary MP level, correcting the EAA profile improved ENU.

In the present study, the infused EAA solutions were not formulated to match a specific EAA profile in the MP supply. Rather, the objective was to meet the requirements, in g/d, of the EAA that became limiting as the dietary CP was reduced. Still, the EAA infusion did alter the profile and made the treatments with EAA infusion closer to the NRC [6] recommendations of Met (2.4% MP), Lys (7.2% MP), and ratio Lys:Met (3:1) compared to the non-infused treatments and the positive control. However, no changes in ENU were observed due to the EAA infusion. Interestingly, the 14.6% CP with EAA infusion treatment had a smaller MUN than the non-infused diet, and this might be a consequence of the better AA profile supplied by the infused treatment. The numerically lower urinary urea N, as a percentage of total urinary N observed for the infused 14.6% CP treatment relative to the non-infused 14.6% CP diet, also corroborates with lower catabolism of AA. However, PUN was not reduced by the EAA infusion at any diet CP level and decreased only with the lowest CP diets.

The effects of the treatments on ENU were somewhat mirrored in the N excretion. Urinary urea N is the main excretory form of excess N [58], and this was also observed in this study, and urinary urea N as a percentage of N was decreased with the reduction in dietary CP.

### 4.2. Mammary Metabolism

The objective of the diet changes and the EAA infusion was to manipulate the amounts and profile of AA reaching the mammary gland and evaluate the effects on mammary metabolism and N efficiency. Dietary CP levels were not as effective as EAA infusion in changing concentrations of arterial AA. This is not surprising considering that the reduction in dietary CP promoted changes from 2.5 and 12.3% in MP supply relative to the positive control, while the amounts of EAA infused corresponded to 3 to 40% of their dietary supply in the infused treatments. For His, Met, and Lys, the change in arterial concentration mirrored the changes in supply. Arterial concentrations of the non-infused group 1 AA, on the other hand, were numerically decreased by the EAA infusion in both diets, suggesting a better utilization of these AA when the more deficient ones were supplied.

The arterial concentration of group 2 AA, however, behaved unexpectedly in the infused 14.6% CP treatment and decreased relative to the positive control even though the predicted supply of digestible EAA from group 2 was the same between both treatments. In fact, the arterial concentration of group 2 AA-N for the infused 14.6% CP treatment was the same as the non-infused 13.2% CP diet, which had a 10% smaller supply.

Interestingly, the concentration of many individual AA, as well as of total AA-N and NEAA-N, were numerically higher for the non-infused 13.2% CP diet than for the non-infused 14.6% CP diet, which might suggest that AA supply from body protein mobilization could have helped the cows on this treatment to meet their EAA requirements and maintain milk protein yield. However, MUN and PUN values for the non-infused 13.2% diet do not support higher body protein mobilization since they were not different from the infused 13.2% CP treatment and were smaller than the other treatments.

Arterial Met concentration was substantially increased by the infusions. As mentioned before, Met is metabolized through the 1 C cycle to donate its methyl group and also to synthesize Cys. Accordingly, the arterial concentration of Cys was greater in the infused treatment compared to the non-infused ones within dietary CP levels. Two other AAs, Ser and Gly, also have roles in the 1 C metabolism cycle [59]. Serine is a precursor for phosphatidylserine, which through a series of steps, receives a methyl group from SAM and is converted to phosphatidylcholine. Serine is also necessary for the conversion of homocysteine to Cys and can be a methyl donor to convert homocysteine back to Met through the folate cycle. Glycine, in turn, can be a methyl group receptor from SAM to be converted to sarcosine, a form of storage of methyl groups when there is an excess supply [59]. Indeed, the arterial concentration of Ser was significantly decreased, and Gly was numerically decreased by EAA infusion relative to the non-infused treatments within dietary CP level. Higher Met and Cys and lower Ser and Gly arterial concentrations in the infused treatments were more pronounced in the infused 13.2% CP treatment, which might indicate higher activity of the 1 C cycle and corroborated the hypothesis that the increase in milk fat yield for the infused 13.2% CP treatment was caused by a greater supply of VLDL to the mammary gland.

The steep accumulation of Met with the infusions suggested it was provided in excess of the requirement [60,61,62]. Change in plasma concentration of EAA as supply increases has been proposed as a method of identifying limiting EAA. However, a recent study evaluated this hypothesis with a large dataset from the literature and concluded that there was no evidence that EAA requirements are reflected in blood plasma concentrations [63].

Numerical differences in mammary total AA-N uptake agree with the numerical differences in milk protein yield. In fact, the numerical increase in milk protein yield for the infused 13.2% CP treatment relative to the non-infused 13.2% CP diet (82 g/d) was the exact same amount observed by Haque et al. [13] and greater than the 68.5 g/d reported by Haque et al. [11] with EAA abomasal infusion, both of which detected as statistical significance. To synthesize the 82 g/d (+8.4% in the present trial vs. 7.5% in Haque et al., [13] more milk protein, the mammary gland increased total AA-N uptake by 16% in our trial and only 7% in Haque et al., [13]. However, our calculations for total AA uptake do not include Arg since it was not analyzed in the plasma, and Arg contributes with four N for the synthesis of NEAA and is an important Pro precursor [37]. Indeed, Haque et al. [13] observed a 13.6% increase in Arg uptake by the mammary gland in their infused treatments.

The fact that AA mammary uptake was not related to arterial AA concentration confirms previous reports that uptake is controlled by intracellular demand [64]. The mammary gland can regulate the cellular AA supply by either altering mammary blood flow or tissue affinity [14]. Clearance rates for His, Met, Phe, Ser, Thr, and Tyr were affected by the treatments. For Met and His, the clearance rate was decreased in the infused treatments, which explains the similar mammary uptakes despite higher arterial concentrations. The clearance rate for Phe, Ser, Thr, and Tyr behaved in the opposite way and increased for the infused treatments, in which arterial concentration had decreased. Interestingly, the substantial increases observed for Phe and Thr in the infused 13.2% CP diet might suggest that these two AAs have become most deficient once the other EAA were provided by the infusion.

Besides the regulation of AA uptake by the mammary gland, the intra-mammary metabolism of each AA can be altered by the need for milk protein synthesis and AA availability. The intra-mammary AA metabolism was inferred by the ratio of mammary AA uptake to milk AA output. The ratio for total AA-N gives the N balance across the mammary gland, and it is expected to be one when all the AA are accounted for. However, as mentioned before, plasma Arg concentration was not analyzed, and Arg contributes four N to the balance, much of it used for Pro synthesis [37]. Therefore, it was expected that the ratio for total AA-N would be smaller than one, as it was in all treatments except infused 13.2% CP, suggesting that less Arg-N was used to synthesize other AA in this treatment. Group 1 AA-N ratio was not different from one, which was expected since they are not metabolized in the mammary gland [37]. Group 2 AA-N ratio was greater than one for all treatments, which also agrees with their known utilization for purposes other than milk protein synthesis in the mammary gland, such as synthesis of NEAA and energy production [37]. However, there was no difference in group 2 AA-N with the AA supplies on the different treatments, while it has been reported in the literature that increased EAA supply to the mammary gland increases the ratio for group 2 AA [37,65]. The mammary gland seems to have a preference for synthesizing NEAA from group 2 AA, even when the supply of NEAA is increased [65]. In agreement, the NEAA-N ratio was smaller than one for all treatments.

## 5. Conclusions

The reduction of dietary protein levels associated with EAA supplementation proved to be a nutritional strategy with a positive effect on N utilization efficiency, as it preserved cow performance, maintaining similar milk production and milk protein synthesis while reducing urinary N excretion. Furthermore, the mammary gland was able to absorb the same amount of AA, despite its lower arterial concentrations, demonstrating that there was plasticity in mammary metabolism to maintain protein synthesis. Finally, N efficiency was not fully explained by changes in mammary metabolism and was likely driven by lower extramammary catabolism as AA supply was reduced.

## Figures and Tables

**Table 1:** Nutrient composition of main dietary ingredients ^1^.

	Alfalfa Silage	Corn Silage	HMSC	SBM	CM	Soy Hulls
DM ^2^, %	38.2	37.5	82.2	91.8	91.5	91.4
CP, % DM	20.7	7.6	8.0	51.6	39.3	10.9
NDF, % DM	39.8	43.0	4.7	8.8	28.0	67.4
ADF, % DM	31.1	25.3	1.3	5.2	19.6	47.3
NDICP, % DM	2.0	1.1	0.3	3.2	5.2	4.0
ADICP, % DM	0.9	0.3	0.1	0.1	2.1	0.9
Ash, % DM	6.1	4.0	1.4	6.5	8.2	4.1
**Amino acids, % CP**						
Alanine	8.00	8.74	8.17	na ^3^	na	4.21
Arginine	2.00	1.72	2.72	7.25	5.84	4.48
Aspartate	4.99	7.72	6.25	na	na	8.86
Cysteine	1.01	0.65	1.87	1.53	2.42	1.74
Glutamate	10.42	6.53	16.41	na	na	10.08
Glycine	3.79	4.22	3.73	na	na	8.40
Histidine	1.54	1.29	2.05	2.67	2.83	2.48
Isoleucine	3.35	4.40	3.36	4.44	3.76	3.54
Leucine	8.15	6.96	11.43	7.50	6.72	6.19
Lysine	2.28	3.78	2.62	6.26	5.40	6.56
Methionine	1.49	1.36	2.08	1.39	1.91	1.08
Phenylalanine	3.40	4.26	4.33	5.00	3.90	3.77
Proline	6.18	5.12	8.46	na	na	5.07
Serine	2.84	3.17	4.21	na	na	5.74
Threonine	3.24	2.96	3.35	3.98	4.26	3.54
Tryptophan	na	na	na	1.38	1.35	na
Valine	4.56	5.41	4.54	4.76	4.87	4.37

^1^ HMSC = high-moisture shelled corn; SBM = solvent-extracted soybean meal, CM = canola meal. ^2^ DM = dry matter; CP = crude protein; NDF = neutral detergent fiber; ADF = acid detergent fiber; NDICP = neutral detergent insoluble crude protein; ADICP = acid detergent insoluble crude protein. ^3^ na = not analyzed.

**Table 2:** Composition of experimental diets.

Dietary CP	16.5	15.0	13.5
Ingredients, % DM			
Alfalfa Silage	30.0	30.0	30.0
Corn Silage	30.0	30.0	30.0
High moisture corn	22.9	23.7	24.8
Solvent soybean meal	5.4	3.6	1.7
Canola meal solvent	6.9	4.5	2.2
Soybean hulls	2.4	5.8	8.9
Calcium sulfate	1.36	1.36	1.36
Monocalcium phosphate	0.22	0.22	0.22
Sodium chloride	0.18	0.18	0.18
Magnesium oxide/sulfate	0.50	0.50	0.50
Vitamin-mineral premix ^1^	0.14	0.14	0.14
Nutrient composition, % DM			
CP, % DM	16.1	14.6	13.2
NDF % DM	30.0	31.5	32.8
ADF, % DM	20.0	21.0	21.9
Ash, % DM	4.3	4.2	4.0
NFC ^2^, % DM	48.5	48.6	48.9
Starch ^3^, % DM	26.7	27.3	28.1

^1^ Provided (per kilogram of diet DM): 67 mg of Zn, 62 mg of Mn, 19 mg of Fe, 12 mg of Cu, 1.4 mg of I, 1 mg of Co, 0.3 mg of Se, 6931 IU of vitamin A, 1386 IU of vitamin D, and 33 IU of vitamin E, and 12 mg of monensin. ^2^ NFC = 100 − % NDF − [% CP − (6.25 × % NDIN)/100] − % EE − % ash. ^3^ Calculated from diet formulation assuming 0, 32, 73, 6, 0, and 3% starch for alfalfa silage, corn silage, high moisture corn, soybean meal, canola meal, and soybean hulls.

**Table 3:** NASEM estimates of protein and amino acids supply and balance and energy balance based on actual inputs ^1^.

Dietary CP	16.1	14.6	14.6	13.2	13.2	SEM	Trt
AA Infusion	−	+	−	+	−		*p*-Value
CP, % DM	16.1 ^a^	14.8 ^b^	14.7 ^c^	13.6 ^d^	13.2 ^e^	0.03	<0.01
RDP, % DM	11.7 ^a^	10.6 ^b^	10.6 ^b^	9.6 ^c^	9.6 ^c^	0.02	<0.01
RUP, % DM	4.5 ^a^	4.2 ^b^	4.0 ^c^	4.0 ^c^	3.6 ^d^	0.02	<0.01
MP supply, g/d	2065 ^a^	1918 ^ab^	1917 ^ab^	1882 ^ab^	1741 ^b^	69	<0.01
MP balance, g/d	−51 ^a^	−190 ^b^	−154 ^ab^	−337 ^c^	−337 ^c^	47	<0.01
NEL supply, Mcal/d	41.8	41.4	42.1	43.5	41.9	2.0	0.78
Nel balance, Mcal/d	3.9	4.3	4.7	3.9	4.9	1.3	0.89
Dig. EAA flow, g/d							
Arginine	104 ^a^	92 ^b^	94 ^b^	84 ^bc^	82 ^c^	3.9	<0.01
Histidine	46 ^b^	47 ^b^	41 ^c^	54 ^a^	37 ^c^	1.8	<0.01
Isoleucine	124 ^a^	113 ^ab^	115 ^ab^	107 ^b^	105 ^b^	4.7	<0.01
Leucine	185 ^a^	170 ^ab^	173 ^ab^	162 ^b^	158 ^b^	7.3	<0.01
Lysine	156 ^b^	156 ^b^	144 ^bc^	174 ^a^	131 ^c^	5.8	<0.01
Methionine	47 ^b^	51 ^a^	43 ^bc^	53 ^a^	40 ^c^	1.8	<0.01
Phenylalanine	116 ^a^	106 ^ab^	107 ^ab^	100 ^b^	98 ^b^	4.3	<0.01
Threonine	109 ^a^	100 ^ab^	101 ^ab^	94 ^b^	92 ^b^	4.2	<0.01
Tryptophan	26 ^a^	24 ^abc^	24 ^ab^	22 ^bc^	21 ^c^	1.0	<0.01
Valine	130 ^a^	119 ^ab^	120 ^ab^	121 ^ab^	110 ^b^	5.0	<0.01
EAA	1043 ^a^	979 ^a^	964 ^ab^	972 ^a^	874 ^b^	39.7	<0.01
Other AA	1817 ^a^	1668 ^ab^	1686 ^ab^	1614 ^b^	1532 ^b^	72.1	<0.01
AA efficiency							
Arginine	0.55 ^e^	0.63 ^c^	0.61 ^d^	0.73 ^a^	0.69 ^b^	0.003	<0.01
Histidine	0.91 ^c^	0.87 ^d^	0.99 ^b^	0.80 ^e^	1.09 ^a^	0.010	<0.01
Isoleucine	0.65 ^e^	0.71 ^c^	0.69 ^d^	0.79 ^a^	0.74 ^b^	0.004	<0.01
Leucine	0.75 ^e^	0.81 ^c^	0.79 ^d^	0.90 ^a^	0.84 ^b^	0.004	<0.01
Lysine	0.74 ^c^	0.73 ^c^	0.80 ^b^	0.70 ^d^	0.86 ^a^	0.007	<0.01
Methionine	0.80 ^c^	0.73 ^d^	0.85 ^b^	0.73 ^d^	0.90 ^a^	0.009	<0.01
Phenylalanine	0.61 ^e^	0.67 ^c^	0.65 ^d^	0.75 ^a^	0.70 ^b^	0.004	<0.01
Threonine	0.64 ^e^	0.70 ^c^	0.68 ^d^	0.78 ^a^	0.74 ^b^	0.003	<0.01
Tryptophan	0.86 ^e^	0.95 ^c^	0.93 ^d^	1.08 ^a^	1.01 ^b^	0.005	<0.01
Valine	0.72 ^d^	0.78 ^b^	0.76 ^c^	0.81 ^a^	0.82 ^a^	0.004	<0.01
EAA	0.70 ^d^	0.74 ^c^	0.75 ^c^	0.79 ^b^	0.80 ^a^	0.004	<0.01

^1^ Computed using NASEM [39] model based on actual chemical composition of feeds, formulated ingredient composition of the diets, analyzed AA composition of infusion solutions and measured amounts infused, and actual animal inputs for each cow × period. Means within row followed by different superscripts differ at *p* ≤ 0.10.

**Table 4:** Effect of diet CP levels and AA infusions on dry matter intake, milk yield and composition, plasma urea nitrogen (PUN), and body weight (BW).

Dietary CP	16.1	14.6	14.6	13.2	13.2	SEM	Trt
AA Infusion	−	+	−	+	−		*p*-Value
DMI, kg/d	23.2	23.1	23.7	24.1	23.3	1.2	0.83
Milk yield, kg/d	35.6	34.3	35.7	36.7	34.6	1.5	0.31
ECM, kg/d	31.9 ^ab^	30.7 ^b^	32.4 ^ab^	34.5 ^a^	31.4 ^b^	1.7	0.09
Fat, %	3.50 ^b^	3.46 ^b^	3.56 ^b^	3.79 ^a^	3.57 ^ab^	0.15	0.05
Fat yield, kg/d	1.236 ^b^	1.173 ^b^	1.269 ^ab^	1.383 ^a^	1.222 ^b^	0.087	0.05
Protein, %	2.91	2.96	2.89	2.99	2.93	0.07	0.27
Protein yield, kg/d	1.026	1.009	1.022	1.082	0.998	0.052	0.25
Lactose, %	4.72	4.76	4.80	4.77	4.83	0.08	0.21
Lactose yield, kg/d	1.689	1.628	1.707	1.746	1.669	0.079	0.45
MUN, mg/dL	12.5 ^a^	10.2 ^c^	11.4 ^b^	9.0 ^d^	8.9 ^d^	0.7	<0.01
PUN, mg/dL	12.4 ^a^	12.1 ^a^	11.2 ^a^	8.1 ^b^	8.7 ^b^	0.8	<0.01

Means within row followed by different superscripts differ at *p* ≤ 0.10.

**Table 5:** Effect of diet CP levels and AA infusions on nutrient intake and digestibility, and microbial protein yield.

Dietary CP	16.1	14.6	14.6	13.2	13.2	SEM	Trt
AA Infusion	−	+	−	+	−		*p*-Value
DM digestibility, %	70.8 ^b^	72.6 ^ab^	71.9 ^ab^	74.0 ^a^	73.7 ^a^	0.9	0.06
OM digestibility, %	72.8	74.5	73.5	75.5	75.3	0.9	0.11
CP digestibility, %	66.8	66.5	65.9	66.5	64.2	1.6	0.75
NDF digestibility, %	51.2 ^c^	55.8 ^ab^	53.1 ^bc^	57.9 ^a^	58.4 ^a^	1.5	<0.01
dDM intake, kg/d	16.4	16.8	17.1	17.8	17.1	0.9	0.41
dOM intake, kg/d	15.7	16.1	16.3	17.1	16.4	0.9	0.43
dCP intake, kg/d	2.5 ^a^	2.2 ^ab^	2.3 ^ab^	2.1 ^bc^	2.0 ^c^	0.1	<0.01
dNDF intake, kg/d	3.5 ^d^	4.1 ^bc^	4.0 ^c^	4.6 ^a^	4.5 ^ab^	0.2	<0.01
Microbial yield, g/d	1321	1342	1349	1374	1329	59	0.87

Means within row followed by different superscripts differ at *p* ≤ 0.10.

**Table 6:** Effect of diet CP levels and AA infusions on N balance.

Dietary CP	16.1	14.6	14.6	13.2	13.2	SEM	Trt
AA Infusion	−	+	−	+	−		*p*-Value
N intake ^1^ (NI), g/d	595 ^a^	551 ^ab^	555 ^ab^	537 ^b^	491 ^c^	27	<0.01
Milk N, g/d	161	158	160	170	157	8	0.26
Milk N/NI, %	26.7 ^c^	28.7 ^b^	28.7 ^b^	31.6 ^a^	31.7 ^a^	1.1	<0.01
Urine N (UN), %	0.75 ^a^	0.60 ^bc^	0.61 ^b^	0.53 ^c^	0.57 ^bc^	0.03	<0.01
UN, g/d	115 ^a^	98 ^bc^	108 ^c^	85 ^d^	89 ^cd^	5	<0.01
UN/NI, %	19.6 ^a^	17.8 ^ab^	19.4 ^a^	15.9 ^b^	18.2 ^b^	0.9	0.01
Urine urea N/UN, %	94.1 ^a^	79.5 ^b^	84.2 ^b^	72.3 ^c^	72.6 ^c^	2.2	<0.01
Fecal N, %	2.9	2.9	2.8	2.7	2.9	0.1	0.22
Fecal N, g/d	196	184	190	170	178	11	0.34
Fecal N/NI, %	33.3	32.9	34.1	31.5	35.6	1.6	0.41
Unaccounted N ^2^, %	20.5	20.3	17.9	19.9	14.6	2.3	0.16

^1^ N intake from the diet plus N infused as AA. ^2^ Unaccounted N = 100 − (milk N/NI) − (urine N/NI) − (fecal N/NI). Means within row followed by different superscripts differ at *p* ≤ 0.10.

**Table 7:** Effect of diet CP levels and AA infusions on arterial AA concentrations (µM).

Dietary CP	16.1	14.6	14.6	13.2	13.2	SEM	Trt
AA Infusion	−	+	−	+	−		*p*-Value
Alanine	281.8	255.5	261.0	290.8	279.8	14.8	0.29
Asparagine	16.1	15.1	15.9	15.3	14.8	1.9	0.98
Aspartate	24.9	19.9	21.8	22.1	23.0	3.0	0.61
Cysteine	142.3 ^ab^	145.8 ^ab^	131.4 ^b^	161.2 ^a^	130.2 ^b^	9.4	0.05
Glutamate	156.9	139.6	138.0	140.8	148.2	11.1	0.30
Glutamine	114.2	115.7	116.5	123.1	120.9	10.9	0.93
Glycine	318.1 ^b^	288.6 ^b^	312.1 ^b^	318.3 ^b^	373.7 ^a^	25.1	0.02
Histidine	50.3 ^b^	52.1 ^ab^	36.2 ^bc^	66.6 ^a^	31.6 ^c^	6.6	<0.01
Isoleucine	115.6 ^a^	101.8 ^b^	108.4 ^a^	100.0 ^b^	101.1 ^b^	7.0	0.10
Leucine	120.4	104.8	109.6	113.4	107.6	6.4	0.17
Lysine	117.0 ^ab^	101.7 ^b^	109.4 ^b^	129.8 ^a^	101.5 ^b^	7.4	0.01
Methionine	25.3 ^c^	36.9 ^b^	23.0 ^c^	51.1 ^a^	24.4 ^c^	2.9	<0.01
Phenylalanine	44.7	40.4	43.6	40.0	45.3	3.2	0.47
Proline	82.1	74.1	77.4	79.1	79.7	4.6	0.66
Serine	98.9 ^ab^	81.5 ^c^	96.7 ^b^	92.5 ^bc^	111.8 ^a^	5.7	<0.01
Threonine	90.6	79.5	94.6	84.1	97.0	7.9	0.21
Tryptophan	46.6	41.7	44.6	40.2	44.3	3.5	0.33
Tyrosine	46.9 ^a^	39.6 ^b^	47.1 ^a^	38.3 ^b^	46.5 ^a^	3.2	<0.01
Valine	192.9 ^a^	158.4 ^b^	176.0 ^ab^	156.4 ^b^	166.6 ^b^	11.7	<0.01
Total AA-N ^1^	2472	2347	2286	2499	2396	111	0.41
EAA-N ^1^	1060 ^ab^	1002 ^abc^	954 ^bc^	1083 ^a^	934 ^c^	53	0.02
NEAA-N ^1^	1413	1299	1336	1424	1466	69	0.32
Group 1 AA-N ^2^	444 ^ab^	445 ^ab^	395 ^b^	493 ^a^	400 ^b^	29	0.02
Group 2 AA-N ^2^	663 ^a^	570 ^b^	617 ^ab^	630 ^ab^	580 ^b^	33	0.05

^1^ Total AA-N: sum of all analyzed AA, on N basis; EAA-N: sum of His, Ile, Leu, Lys, Met, Phe, Thr, Trp, and Val, on N basis; NEAA-N: sum of Ala, Asn, Asp, Asn, Cys, Glu, Gln, Gly, Pro, Ser, and Tyr, on N basis. ^2^ Group 1 AA-N: sum of His, Met, Phe+Tyr, Thr, and Trp, on N basis; Group 2 AA-N: sum of Ile, Leu, Lys, and Val, on N basis. Means within row followed by different superscripts differ at *p* ≤ 0.10.

**Table 8:** Effect of diet CP levels and AA infusions on mammary plasma flow (L/h) and mammary gland AA uptake (mmol/h).

Dietary CP	16.1	14.6	14.6	13.2	13.2	SEM	Trt
AA Infusion	−	+	−	+	−		*p*-Value
Plasma flow	802	867	839	819	831	107	0.99
Alanine	26.6	27.2	28.7	32.0	26.3	4.3	0.66
Asparagine	3.30	2.92	2.94	3.60	3.15	0.68	0.90
Aspartate	4.50	4.49	3.47	3.96	4.42	0.73	0.61
Cysteine	3.00	4.71	6.58	4.60	3.84	2.10	0.69
Glutamate	40.7	40.4	37.0	35.6	39.3	3.9	0.73
Glutamine	16.7 ^b^	23.4 ^ab^	23.8 ^ab^	27.2 ^a^	19.2 ^ab^	3.3	0.08
Glycine	1.80	2.37	5.59	3.53	−4.54	5.89	0.69
Histidine	11.8	6.9	7.4	10.8	8.8	3.2	0.45
Isoleucine	24.1	24.1	24.2	23.4	24.0	2.1	1.00
Leucine	36.2	35.6	35.9	36.2	35.1	2.6	0.99
Lysine	38.4	30.2	31.6	41.4	30.3	5.8	0.24
Methionine	9.20	9.44	8.44	8.90	7.93	0.74	0.42
Phenylalanine	14.6	14.0	14.6	14.7	13.9	1.0	0.90
Proline	8.10	6.96	7.68	7.85	7.14	1.11	0.86
Serine	16.8	18.0	15.7	17.9	13.7	2.0	0.40
Threonine	18.2	17.2	16.4	16.5	14.5	1.8	0.34
Tryptophan	4.90	3.61	4.42	3.67	3.41	1.83	0.94
Tyrosine	12.0	12.5	11.9	12.9	12.0	0.9	0.62
Valine	27.7	27.4	28.5	27.4	27.3	2.8	0.99
Total AA-N ^1^	402	405	383	421	361	40	0.36
EAA-N ^1^	252	228	223	248	219	29	0.55
NEAA-N ^1^	150	175	170	177	146	20	0.33
Group 1 AA-N ^2^	95	82	84	92	83	13	0.75
Group 2 AA-N ^2^	165	152	151	166	147	15	0.57

^1^ Total AA-N: sum of all analyzed AA, on N basis; EAA-N: sum of His, Ile, Leu, Lys, Met, Phe, Thr, Trp, and Val, on N basis; NEAA-N: sum of Ala, Asn, Asp, Asn, Cys, Glu, Gln, Gly, Pro, Ser, and Tyr, on N basis. ^2^ Group 1 AA-N: sum of His, Met, Phe+Tyr, Thr, and Trp, on N basis; Group 2 AA-N: sum of Ile, Leu, Lys, and Val, on N basis. Means within row followed by different superscripts differ at *p* ≤ 0.10.

**Table 9:** Effect of diet CP levels and AA infusions on AA ^1^ clearance rate (L/h).

Dietary CP	16.1	14.6	14.6	13.2	13.2	SEM	Trt
AA Infusion ^1^	−	+	−	+	−		*p*-Value
Alanine	230	118	121	106	97	102	0.74
Asparagine	303	230	270	392	272	82	0.56
Aspartate	262	285	287	257	275	52	0.98
Cysteine	22	32	57	31	31	16	0.46
Glutamate	400	447	429	433	431	57	0.95
Glutamine	247	271	282	294	202	40	0.18
Histidine	443 ^ab^	166 ^b^	343 ^ab^	287 ^ab^	518 ^a^	129	0.10
Isoleucine	300	331	325	368	327	40	0.44
Leucine	518	642	560	622	602	72	0.30
Lysine	606	418	579	608	555	116	0.69
Methionine	675 ^a^	368 ^b^	674 ^a^	232 ^b^	591 ^a^	67	<0.01
Phenylalanine	608 ^bc^	700 ^ab^	580 ^bc^	755 ^a^	543 ^c^	74	0.06
Proline	116	106	121	116	107	19	0.93
Serine	224 ^bc^	288 ^ab^	224 ^bc^	327 ^a^	172 ^c^	32	<0.01
Threonine	279 ^a^	294 ^a^	234 ^a^	284 ^a^	201 ^b^	32	0.03
Tryptophan	90	128	109	108	96	43	0.97
Tyrosine	409 ^b^	539 ^b^	424 ^b^	687 ^a^	414 ^b^	67	<0.01
Valine	181	223	209	246	214	29	0.18
Total AA-N ^2^	212	216	220	217	184	24	0.20
EAA-N ^2^	356	316	349	351	342	49	0.94
NEAA-N ^2^	127	156	158	149	117	20	0.13
Group 1 AA-N ^3^	288	233	290	267	287	43	0.73
Group 2 AA-N ^3^	376	394	386	427	362	44	0.50

^1^ Glycine is not presented because its mammary net flux was not different from zero. ^2^ Total AA-N: sum of all analyzed AA, on N basis; EAA-N: sum of His, Ile, Leu, Lys, Met, Phe, Thr, Trp, and Val, on N basis; NEAA-N: sum of Ala, Asn, Asp, Asn, Cys, Glu, Gln, Gly, Pro, Ser, and Tyr, on N basis. ^3^ Group 1 AA-N: sum of His, Met, Phe+Tyr, Thr, and Trp, on N basis; Group 2 AA-N: sum of Ile, Leu, Lys, and Val, on N basis. Means within row followed by different superscripts differ at *p* ≤ 0.10.

**Table 10:** Effect of diet CP levels and AA1 infusions on mammary gland AA uptake:milk AA output ratio.

Dietary CP	16.1	14.6	14.6	13.2	13.2	SEM	Trt
AA Infusion	−	+	−	+	−		*p*-Value
Milk protein yield ^1,2^	497.0	499.4	500.3	520.1	488.3	0.03	0.85
Alanine	1.55 *	1.72 *	1.78 *	1.94 *	1.69 *	0.26	0.72
Asparagine	0.24 *	0.19 *	0.23 *	0.27 *	0.23 *	0.05	0.84
Aspartate	0.33 *	0.36 *	0.30 *	0.34 *	0.40 *	0.05	0.51
Cysteine	0.93	1.71	2.69 *	1.77	1.46	0.81	0.49
Glutamate	1.12	1.15	1.03	0.96	1.12	0.09	0.41
Glutamine	0.65 *	0.83	0.80 *	0.92	0.66 *	0.11	0.23
Histidine	1.43 *	0.76	1.02	1.00	1.23	0.33	0.34
Isoleucine	1.22 *	1.17 *	1.23 *	1.25 *	1.25 *	0.05	0.53
Leucine	1.12 *	1.08 *	1.09 *	1.12 *	1.10 *	0.02	0.40
Lysine	1.57 *	1.14	1.32 *	1.60 *	1.27 ^†^	0.20	0.21
Methionine	1.09 *^a^	1.03^ab^	0.98^b^	1.02 ^ab^	0.96 ^b^	0.04	0.07
Phenylalanine	1.12 *	1.07 *	1.09 *	1.09 *	1.09 *	0.02	0.34
Proline	0.22 *	0.18 *	0.21 *	0.20 *	0.19 *	0.02	0.62
Serine	0.65 *^abc^	0.68 *^ab^	0.60 *^bc^	0.74 *^a^	0.56 *^c^	0.46	0.03
Threonine	1.10	1.06	1.03	1.09	0.92	0.07	0.20
Tryptophan	1.46	1.28	1.38	1.18	1.24	0.52	0.99
Tyrosine	0.89 *	0.94 *	0.89 *	0.92 *	0.91 *	0.02	0.35
Valine	1.11 ^†^	1.09	1.15 *	1.20 *	1.14 *	0.07	0.72
Total AA-N ^3^	0.92 ^†^	0.92 ^†^	0.91 ^†^	0.98	0.84 *	0.06	0.12
EAA-N ^3^	1.29 *	1.11	1.18 *	1.30 *	1.14 ^†^	0.12	0.40
NEAA-N ^3^	0.68 *	0.73 *	0.73 *	0.74 *	0.63 *	0.06	0.39
Group 1 AA-N ^4^	1.05	0.77	0.87	1.04	0.90	0.17	0.51
Group 2 AA-N ^4^	1.31 *	1.16 ^†^	1.23 *	1.27 *	1.20 *	0.09	0.53

^1^ Glycine is not presented because its mammary net flux was not different from zero. ^2^ Milk protein yield in the milking immediately following the blood sampling. ^3^ Total AA-N: sum of all analyzed AA, on N basis; EAA-N: sum of His, Ile, Leu, Lys, Met, Phe, Thr, Trp, and Val, on N basis; NEAA-N: sum of Ala, Asn, Asp, Asn, Cys, Glu, Gln, Gly, Pro, Ser, and Tyr, on N basis. ^4^ Group 1 AA-N: sum of His, Met, Phe+Tyr, Thr, and Trp, on N basis; Group 2 AA-N: sum of Ile, Leu, Lys, and Val, on N basis. * Different from one at *p* < 0.05; ^†^ tendency at *p* < 0.10. Means within row followed by different superscripts differ at *p* ≤ 0.10.

## Data Availability

Not applicable.

## References

[1] Dijkstra J., Reynolds C.K., Kebreab E., Bannink A., Ellis J.L., France J., van Vuuren A.M., Oltjen W.J., Kebreab E., Lapierre H. (2013). Challenges in ruminant nutrition: Towards minimal nitrogen losses in cattle. Energy and Protein Metabolism and Nutrition in Sustainable Animal Production.

[2] Huhtanen P., Hristov A.N. (2009). A meta-analysis of the effects of dietary protein concentration and degradability on milk protein yield and milk N efficiency in dairy cows. J. Dairy Sci..

[3] Arriola Apelo S.I., Knapp J.R., Hanigan M.D. (2014). Invited review: Current representation and future trends of predicting amino acid utilization in the lactating dairy cow. J. Dairy Sci..

[4] Hanigan M.D., Crompton L.A., Reynolds C.K., Wray-Cahen D., Lomax M.A., France J. (2004). An integrative model of amino acid metabolism in the liver of the lactating dairy cow. J. Theor. Biol..

[5] Hanigan M.D., Reynolds C.K., Humphries D.J., Lupoli B., Sutton J.D. (2004). A model of net amino acid absorption and utilization by the portal-drained viscera of the lactating dairy cow. J. Dairy Sci..

[6] NRC (2001). Nutrient Requirements of Dairy Cattle.

[7] Doepel L., Pacheco D., Kennelly J.J., Hanigan M.D., López I.F., Lapierre H. (2004). Milk protein synthesis as a function of amino acid supply. J. Dairy Sci..

[8] Danes M.A.C., Hanigan M.D., Arriola Apelo S.I., Dias J.D.L., Wattiaux M.A., Broderick G.A. (2020). Post-ruminal supplies of glucose and casein, but not acetate, stimulate milk protein synthesis in dairy cows through differential effects on mammary metabolism. J. Dairy Sci..

[9] Pszczolkowski V.L., Zhang J., Pignato K.A., Meyer E.J., Kurth M.M., Lin A., Arriola Apelo S.I. (2020). Insulin potentiates essential amino acids effects on mechanistic target of rapamycin complex 1 signaling in MAC-T cells. J. Dairy Sci..

[10] Olmos Colmenero J.J., Broderick G.A. (2006). Effect of dietary crude protein concentration on milk production and nitrogen utilization in lactating dairy cows. J. Dairy Sci..

[11] Haque M.N., Rulquin H., Andrade A., Faverdin P., Peyraud J.L., Lemosquet S. (2012). Milk protein synthesis in response to the provision of an “ideal” amino acid profile at 2 levels of metabolizable protein supply in dairy cows. J. Dairy Sci..

[12] Nahm K.H. (2002). Efficient feed nutrient utilization to reduce pollutants in poultry and swine manure. Crit. Rev. Environ. Sci. Technol..

[13] Haque M.N., Guinard-Flament J., Lamberton P., Mustière C., Lemosquet S. (2015). Changes in mammary metabolism in response to the provision of an ideal amino acid profile at 2 levels of metabolizable protein supply in dairy cows: Consequences on efficiency. J. Dairy Sci..

[14] Bequette B.J., Hanigan M.D., Calder A.G., Reynolds C.K., Lobley G.E., MacRae J.C. (2000). Amino acid exchange by the mammary gland of lactating goats when histidine limits milk production. J. Dairy Sci..

[15] Cantalapiedra-Hijar G., Ortigues-Marty I., Lemosquet S. (2015). Diets rich in starch improve the efficiency of amino acids use by the mammary gland in lactating Jersey cows. J. Dairy Sci..

[16] Curtis R.V., Kim J.J.M., Doelman J., Cant J.P. (2018). Maintenance of plasma branched-chain amino acid concentrations during glucose infusion directs essential amino acids to extra-mammary tissues in lactating dairy cows. J. Dairy Sci..

[17] Nichols K., Bannink A., Doelman J., Dijkstra J. (2019). Mammary gland metabolite utilization in response to exogenous glucose or long-chain fatty acids at low and high metabolizable protein levels. J. Dairy Sci..

[18] Gressley T.F., Reynal S.M., Colmenero J.O., Broderick G.A., Armentano L.E. (2006). Technical note: Development of a tool to insert abomasal infusion lines into dairy cows. J. Dairy Sci..

[19] AOAC (Association of Official Analytical Chemists) (1990). Official Methods of Analysis.

[20] AOAC (1980). Official Methods of Analysis.

[21] Van Soest P.J., Robertson J.B., Lewis B.A. (1991). Methods for dietary fiber, neutral detergent fiber, and nonstarch polysaccharides in relation to animal nutrition. J. Dairy Sci..

[22] Hintz R.W., Mertens D.R., Albrecht K.A. (1996). Effects of sodium sulfite on recovery and composition of detergent fiber and lignin. J. AOAC Int..

[23] Licitra G., Hernandez T.M., Van Soest P.J. (1996). Standardization of procedures for nitrogen fractionation of ruminant feeds. Anim. Feed Sci. Technol..

[24] AOAC International (1995). Official Methods of Analysis.

[25] European Commission (2009). 2009/150/EC Commission regulation laying down the methods of sampling and analysis for the official control of feed. Off. J. Eur. Union L..

[26] Tyrrell H.F., Reid J.T. (1965). Prediction of the energy value of cow’s milk. J. Dairy Sci..

[27] Krause K.M., Combs D.K., Beauchemin K.A. (2003). Effects of increasing levels of refined cornstarch in the diet of lactating dairy cows on performance and ruminal pH. J. Dairy Sci..

[28] Broderick G.A., Clayton M.K. (1997). A statistical evaluation of animal and nutritional factors influencing concentrations of milk urea nitrogen. J. Dairy Sci..

[29] Valadares R.F.D., Broderick G.A., Valadares Filho S.C., Clayton M.K. (1999). Effect of replacing alfalfa silage with high moisture corn on ruminal protein synthesis estimated from excretion of total purine derivatives. J. Dairy Sci..

[30] Vogels G.D., Van der Drift C. (1970). Differential analyses of glyoxylate derivatives. Anal. Biochem..

[31] Chen X.B., Gomes M.J. (1992). Estimation of Microbial Protein Supply to Sheep and Cattle Based on Urinary Excretion of Purine Derivatives—An Overview of the Technical Details.

[32] Vagnoni D.B., Broderick G.A., Clayton M.K., Hatfield R.D. (1997). Excretion of purine derivatives by holstein cows abomasally infused with incremental amounts of purines. J. Dairy Sci..

[33] Emery R.S., Brown L.D., Bell J.W. (1965). Correlation of milk fat with dietary and metabolic factors in cows fed restricted roughage rations supplemented with magnesium oxide or sodium bicarbonate. J. Dairy Sci..

[34] Chaney A.L., Marbach E.P. (1962). Modified reagents for determination of urea and ammonia. Clin. Chem..

[35] Calder A.G., Garden K.E., Anderson S.E., Lobley G.E. (1999). Quantitation of blood and plasma amino acids using isotope dilution electron impact gas chromatography/mass spectrometry with U-13C amino acids as internal standards. Rapid Commun. Mass Spectrom..

[36] Cant J.P., DePeters E.J., Baldwin R.L. (1993). Mammary amino acid utilization in dairy cows fed fat and its relationship to milk protein depression. J. Dairy Sci..

[37] Lapierre H., Lobley G.E., Doepel L., Raggio G., Rulquin H., Lemosquet S. (2012). Triennial Lactation Symposium: Mammary metabolism of amino acids in dairy cows. J. Anim. Sci..

[38] Hanigan M.D., France J., Wray-cahen D., Beever D.E., Lobley G.E., Reutzel L., Smith E.N. (1998). Alternative models for analyses of liver and mammary transorgan metabolite extraction data. Br. J. Nutr..

[39] NASEM (National Academies of Sciences, Engineering, and Medicine) (2021). Nutrient Requirements of Dairy Cattle.

[40] Zhao S., Min L., Zheng N., Wang J. (2019). Effect of heat stress on bacterial composition and metabolism in the rumen of lactating dairy cows. Animals.

[41] Lee C., Hristov A.N., Cassidy T.W., Heyler K.S., Lapierre H., Varga G.A., de Veth M.J., Patton R.A., Parys C. (2012). Rumen protected lysine, methionine, and histidine increase milk protein yield in dairy cows fed a metabolizable protein-deficient diet. J. Dairy Sci..

[42] Zhao K., Liu W., Lin X.Y., Hu Z.Y., Yan Z.G., Wang Y., Shi K.R., Liu G.M., Wang Z.H. (2019). Effects of rumen-protected methionine and other essential amino acid supplementation on milk and milk component yields in lactating Holstein cows. J. Dairy Sci..

[43] Giallongo F., Hristov A.N., Oh J., Frederick T., Weeks H., Werner J., Lapierre H., Patton R.A., Gehman A., Parys C. (2015). Effects of slow-release urea and rumen-protected methionine and histidine on performance of dairy cows. J. Dairy Sci..

[44] Lee C., Giallongo F., Hristov A.N., Lapierre H., Cassidy T.W., Heyler K.S., Varga G.A., Parys C. (2015). Effect of dietary protein level and rumen-protected amino acid supplementation on amino acid utilization for milk protein in lactating dairy cows. J. Dairy Sci..

[45] Reynolds C.K., Kristensen N.B. (2008). Nitrogen recycling through the gut and the nitrogen economy of ruminants: An asynchronous symbiosis. J. Anim. Sci..

[46] Arriola Apelo S.I., Bell A.L., Estes K., Ropelewski J., de Veth M.J., Hanigan M.D. (2014). Effects of reduced dietary protein and supplemental rumen-protected essential amino acids on the nitrogen efficiency of dairy cows. J. Dairy Sci..

[47] Arriola Apelo S.I., Singer L.M., Lin X.Y., McGilliard M.L., St-Pierre N.R., Hanigan M.D. (2014). Isoleucine, leucine, methionine, and threonine effects on mammalian target of rapamycin signaling in mammary tissue. J. Dairy Sci..

[48] Appuhamy J.A., Knoebel N.A., Nayananjalie W.A., Escobar J., Hanigan M.D. (2012). Isoleucine and leucine independently regulate mTOR signaling and protein synthesis in MAC-T cells and bovine mammary tissue slices. J. Nutr..

[49] Saxton R.A., Knockenhauer K.E., Wolfson R.L., Chantranupong L., Pacold M.E., Wang T., Schwartz T.U., Sabatini D.M. (2016). Structural basis for leucine sensing by the Sestrin2-mTORC1 pathway. Science.

[50] Sabatini D.M. (2017). Twenty-five years of mTOR: Uncovering the link from nutrients to growth. Proc. Natl. Acad. Sci. USA.

[51] Lemosquet S., Raggio G., Lapierre H., Guinard-Flament J., Rulquin H. (2007). Effects of protein supply on whole body glucose rate of appearance and mammary gland metabolism of energy nutrients in ruminants. ISEP—2nd International Symposium on Energy and Protein Metabolism and Nutrition.

[52] Greenberg D.M. (1963). Biological Methylation. Adv. Enzymol. Rel. Subj. Bioch..

[53] Wang C., Liu H.Y., Wang Y.M., Yang Z.Q., Liu J.X., Wu Y.M., Yan T., Ye H.W. (2010). Effects of dietary supplementation of methionine and lysine on milk production and nitrogen utilization in dairy cows. J. Dairy Sci..

[54] Osorio J.S., Ji P., Drackley J.K., Luchini D., Loor J.J. (2013). Supplemental Smartamine M or MetaSmart during the transition period benefits postpartal cow performance and blood neutrophil function. J. Dairy Sci..

[55] Li S., Hosseini A., Danes M., Jacometo C., Liu J., Loor J.J. (2016). Essential amino acid ratios and mTOR affect lipogenic gene networks and miRNA expression in bovine mammary epithelial cells. J. Anim. Sci. Biotechnol..

[56] Aguerre M.J., Wattiaux M.A., Hunt T., Larget B.R. (2010). Effect of dietary crude protein on ammonia-N emission measured by herd nitrogen mass balance in a freestall dairy barn managed under farm-like conditions. Animal.

[57] Rulquin H., Raggio G., Lapierre H., Lemosquet S. (2007). Relationship between intestinal supply of essential amino acids and their mammary metabolism in the lactating dairy cow. Energy and Protein Metabolism and Nutrition.

[58] Castillo A.R., Kebreab E., Beever D.E., France J. (2001). A review of efficiency of nitrogen utilisation in lactating dairy cows and its relationship with environmental pollution. J. Anim. Feed Sci..

[59] Benevenga N.J. (1974). Toxicities of Methionine and Other Amino Acids. J. Agr. Food Chem..

[60] Broderick G.A., Satter L.D., Harper A.E. (1974). Use of plasma amino acid concentration to identify limiting amino acids for milk production. J. Dairy Sci..

[61] Raggio G., Lemosquet S., Lobley G.E., Rulquin H., Lapierre H. (2006). Effect of casein and propionate supply on mammary protein metabolism in lactating dairy cows. J. Dairy Sci..

[62] Letelier P., Zanton G.I., Dorea J.R.R., Wattiaux M.A. (2022). Plasma essential amino acid concentration and profile are associated with performance of lactating dairy cows as revealed through meta-analysis and hierarchical clustering. J. Dairy Sci..

[63] Patton R.A., Hristov A.N., Parys C., Lapierre H. (2015). Relationships between circulating plasma concentrations and duodenal flows of essential amino acids in lactating dairy cows. J. Dairy Sci..

[64] Hanigan M.D., France J., Crompton L.A., Bequette B.J. (2000). Evaluation of a representation of the limiting amino acid theory for milk protein synthesis. Modelling Nutrient Utilization in Farm Animals.

[65] Doepel L., Lapierre H. (2010). Changes in production and mammary metabolism of dairy cows in response to essential and nonessential amino acid infusions. J. Dairy Sci..

